# Factors Influencing the Prevalence of Hyperpigmented Melanistic Lesions in Smallmouth Bass *Micropterus dolomieu* in the Susquehanna River Basin, Pennsylvania

**DOI:** 10.1111/jfd.14033

**Published:** 2024-10-23

**Authors:** Megan K. Schall, Geoffrey D. Smith, Vicki S. Blazer, Heather L. Walsh, Tyler Wagner

**Affiliations:** ^1^ Biological Sciences, Penn State Hazleton 76 University Drive Hazleton Pennsylvania USA; ^2^ Pennsylvania Fish and Boat Commission, Division of Fisheries Management Bellefonte Pennsylvania USA; ^3^ U.S. Geological Survey, Eastern Ecological Science Center – Leetown Research Laboratory Kearneysville West Virginia USA; ^4^ U.S. Geological Survey, Pennsylvania Cooperative Fish and Wildlife Research Unit The Pennsylvania State University University Park Pennsylvania USA

**Keywords:** fish disease modelling, fish health monitoring, hyperpigmented melanistic lesions, smallmouth bass

## Abstract

Hyperpigmented melanistic lesions (HPMLs) are a visual anomaly documented on the skin of smallmouth bass *Micropterus dolomieu* in the Susquehanna River Basin, Pennsylvania and in numerous other geographical locations. Currently, there is a lack of information on environmental and fish characteristics that may influence the prevalence of HPMLs associated with a recently described *Adomavirus*. The goal of this study was to understand potential drivers associated with HPMLs in socioeconomically and ecologically important riverine smallmouth bass populations. A total of 16,220 smallmouth bass were collected and examined for HPMLs between 2012 and 2022 in the Susquehanna River Basin. Overall, HPMLs were documented on 2.9% of fish collected. The interaction between temperature and fish size suggested differing relationships between shorter and longer fish with respect to temperature. Predicted probability of HPML prevalence ranged from 1.1% (95% CI = 0.3, 3.2) at 4°C to 0.01% (CI = 0.00, 0.04) at 26°C for an age‐0 (125 mm) fish. In contrast, predicted probability of HPML prevalence ranged from 10.5% (95% CI = 5.8, 18.9) at 4°C to 0.8% (CI = 0.4, 1.5) at 26°C for an adult (322 mm) fish. Overall, HPMLs were more common in longer fish during cooler temperature periods which also corresponds to key life history periods for smallmouth bass (e.g., pre‐spawn and overwintering) and could represent different exposure histories for juvenile and adult fish.

## 1 Introduction

Smallmouth bass *Micropterus dolomieu* support important recreational fisheries throughout the United States. In the Susquehanna River Basin, Pennsylvania, smallmouth bass have been long regarded as a renowned recreational fishery (Arway and Smith 2013). However, smallmouth bass populations in this system have a history of mortality events among juveniles and disease in both juvenile and adult fish that have likely contributed to population fluctuations over the past two decades (Smith et al. 2015; Shull and Pulket 2015; Schall et al. 2020) and heightened sensitivity to disease among anglers, the public, and fisheries managers. Most recently, populations have approached pre‐disease catch rates and have demonstrated signs of population recovery (i.e., lower natural mortality rates; Li et al. 2018; Schall et al. 2020). Yet, population declines in the early to mid‐2000s resulted in an unprecedented amount of research focusing on infectious disease including viral (largemouth bass virus; Boonthai et al. 2018), parasitic (Walsh et al. 2018; Schall et al. 2018) and bacterial pathogens (Starliper et al. 2013; Walsh et al. 2018), as well as the sub‐lethal effects of exposure to endocrine disrupting chemicals (Blazer, Iwanowicz, et al. 2014; Li et al. 2020), mercury (Blazer et al. 2023) and other environmental stressors. During these extensive population surveys, adult and young‐of‐year smallmouth bass were often collected with anomalies on the skin, including multiple types of lesions associated with differing underlying etiology and disease agents including many opportunistic pathogens (Blazer et al. 2010; Starliper et al. 2013; Walsh et al. 2018). The visual appearance of potentially unhealthy fish has generated great public interest and could contribute to the negative perception of a recreational sport fishery appearing in poor overall health (Arway and Smith 2013).

Hyperpigmented melanistic lesions (HPMLs) are an anomaly encountered on the skin surface of smallmouth bass from the Susquehanna River Basin and elsewhere. These lesions appear as brown or black, non‐raised pigmented areas, referred to as “blotchy bass syndrome” or “black blotch bass” when initially observed in the 1980s from largemouth bass *Micropterus nigricans* in the Hudson River (Carlson 1989). They have also been reported in both largemouth and smallmouth bass from tributaries of the Great Lakes (Blazer, Mazik et al. 2014). Awareness was raised after HPMLs were encountered throughout the Susquehanna River Basin by anglers and during routine management surveys and fish health assessments beginning in the early 2010s. Histological examination of the lesions identified proliferation of melanin‐producing melanophores in the dermis and/or epidermis (Blazer et al. 2020; Young 2018). Hyperpigmented melanistic lesions were encountered on 5% and 1% of smallmouth bass from the Susquehanna River mainstem and Juniata River, a major tributary of the Susquehanna River, respectively, during routine black bass surveys with prevalence ranging as high as 20% during a single sampling event (Blazer et al. 2020). Anecdotal reports and preliminary data suggested that HPMLs were more commonly encountered in the early spring and late fall indicating that seasonality and temperature may be factors associated with their development (Blazer et al. 2020). Yet, a laboratory study on smallmouth bass with and without HPMLs failed to demonstrate a clear temperature association; however, HPMLs did change in appearance and fish both gained and lost lesions throughout the study duration (Young et al. 2019). Subsequent molecular and transcriptomic studies of HPMLs from smallmouth bass revealed the presence of a novel viral helicase most similar to recently described adomavirus belonging to Adomaviridae (Blazer et al. 2020). This suggested that the presence of HPMLs could be associated with a response to the virus. Several genes were also identified with differential expression in HPMLs when compared to normal skin, including seven related to melanin production, and three others involved in aspects of cell growth, signalling, and immune response (Blazer et al. 2020).

Despite recent advances in our knowledge of HPMLs on smallmouth bass, there is a general lack of understanding regarding this viral infection including localization of viral particles in HPMLs, and association of fish characteristics (age/length, immune status, reproductive stage) and environmental drivers (temperature, chemical contaminants) with the prevalence of HPMLs. Additionally, previous studies have been limited in spatiotemporal scope, preventing inferences on long‐term patterns and/or investigation into spatial and temporal factors that could influence viral infection and HPML prevalence. Hyperpigmented melanistic lesions have been tracked by the state fishery management agency in Pennsylvania (the Pennsylvania Fish and Boat Commission) during routine black bass surveys in the Susquehanna and Juniata rivers dating back to 2012. The objectives for this study were to (1) use a long‐term dataset to evaluate spatial and temporal differences in HPML prevalence, including relationships between HPML prevalence and both environmental (e.g., temperature) and fish characteristics (e.g., fish length) and (2) investigate location of viral particles in association with HPMLs. Given that HPMLs are a visual health abnormality associated with a viral infection, it is our goal to not only better understand factors that could contribute to occurrence but also consider underlying mechanisms related to infection and smallmouth bass health.

## 2 Methods

### 2.1 Study Area

**FIGURE 1:**
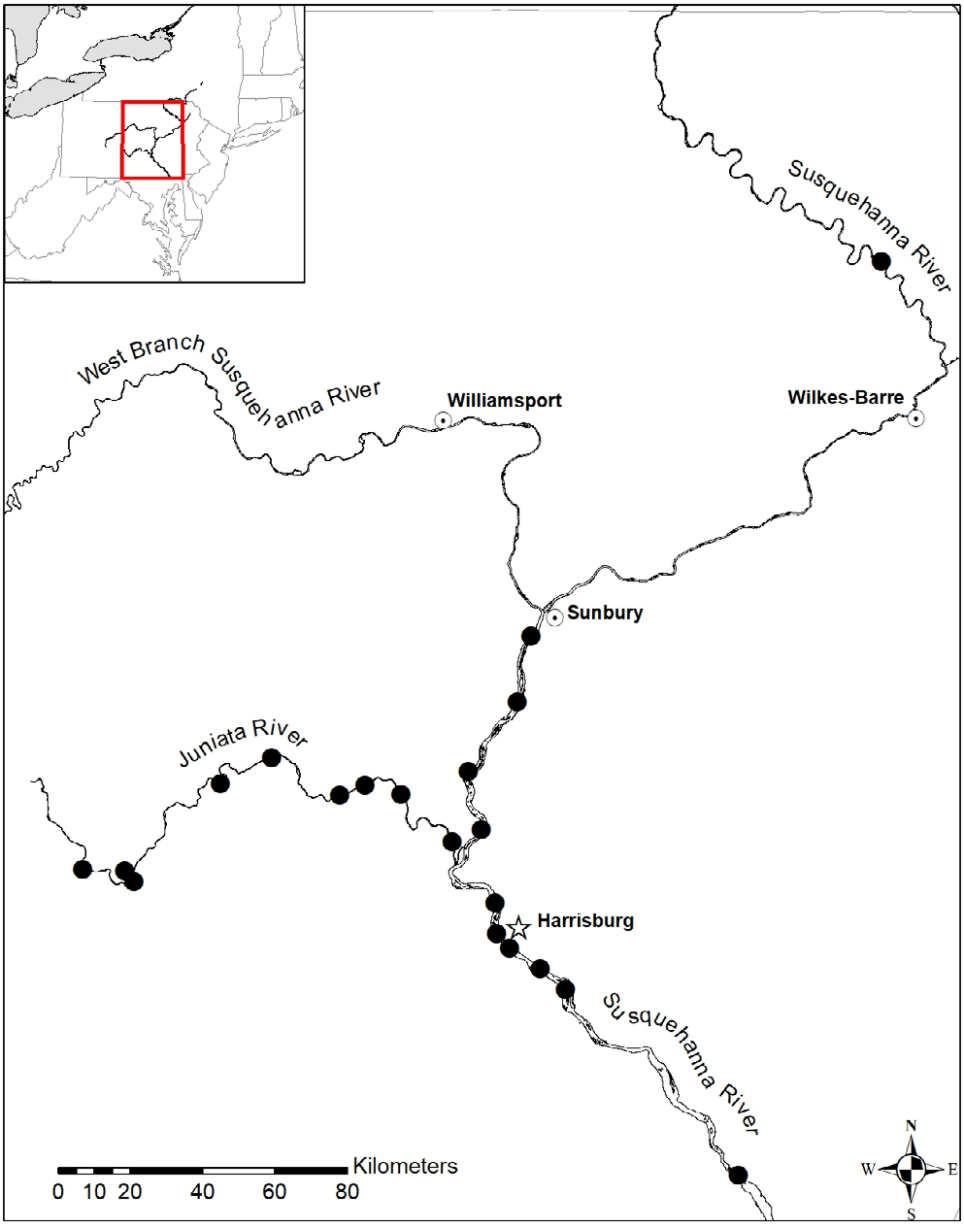
The twenty sampling sites in the Susquehanna River Basin, Pennsylvania used to investigate the prevalence of hyperpigmented melanistic lesions (HPMLs) in smallmouth bass between 2012 and 2022. Black circles represent sampling locations, clear circles with a black center dot represent major cities for geographical references, and the star represents the capital of Pennsylvania (Harrisburg).

The Susquehanna River Basin is the largest contributor of freshwater to the Chesapeake Bay and covers over 71,000 km^2^ across New York, Pennsylvania, and Maryland. Routine black bass (primarily smallmouth bass) surveys occurred at least once at a total of 20 sites located throughout the mainstem of the Susquehanna River, Pennsylvania between the Conowingo Dam in Maryland and the New York state border, and in the Juniata River; a tributary of the Susquehanna River (Figure 1). The Susquehanna River proper and Juniata River represent regions with prior smallmouth bass health concerns and where HPMLs were previously documented (Blazer et al. 2020).

### 2.2 Fish Collection

Adult smallmouth bass were collected as part of routine surveys between 2012 and 2022. Surveys occurred in March, April, May, June, September, October or November and aligned with key life history points for smallmouth bass (i.e., pre [March–April] and post [June–September] spawning and preparation for overwintering and recrudescence [October–November]). Fish were collected using boat‐mounted electrofishers with pulsed direct current (60 Hz, 25%–30% duty cycle). Collected fish were held in live wells on the boat prior to visual inspection. The total length (TL) of each fish was measured and recorded to the nearest millimetre (mm) or binned into 25‐mm TL groups depending on the survey goal. Each fish was visually inspected for HPMLs, which were characterised as being present or absent. Biologists handling and assessing fish in the field were provided training on how to differentiate HPMLs from seasonal coloration changes due to spawning, branding from electrofishing or other discolorations of the fish. There was no attempt to qualify or quantify the intensity of melanistic areas or spatial coverage on individual fish. Water temperature measurements (°C) were taken along with each collection event. Coinciding with routine sampling, a subsample of adult smallmouth bass were euthanized during focused fish health surveys using a lethal dose (350 mg/L) of Tricaine methanesulphonate (MS222, Syndel, Ferndale, WA). Pieces of both normal and affected skin were preserved in PAXgene (Qiagen, Hilden, Germany) according to manufacturer's protocol for localization of viral nucleic acids associated with HPMLs.

RNAscope methodology was developed to identify the location (i.e., target tissue and/or cells) of the viral protein gene *M. dolomieu* adomavirus 1 LO8 gene (MdA1 LO8; NCBI Accession No. MZ673484.1) as evidence for viral infection associated with HPMLs. Tissues were processed for histopathology, embedded into paraffin, and sectioned at 5 μm. RNAscope (Advanced Cell Diagnostics, Newark, California) was used to observe the localization of the target adomavirus virion protein gene, adenain (LO8). Selected sections with HPMLs were deparaffinised with three changes of Pro‐Par clearant (Anatech Ltd., Michigan) for 5 min each and rehydrated with a graded ethanol series of 100%, 95%, 80%, 70%, and 50% for 3 min each and air dried. RNAscope was conducted according to the manufacturer's protocols for the RNAscope Multiplex Fluorescent Reagent Kit v2 Assay. One change in the assay was that the RNAscope Protease Plus incubation step was omitted because early trials of this assay with PAXgene preserved samples revealed significant tissue destruction and a lack of positive signal. Next, the target probe for MdA1 LO8 was prepared by warming for 10 min at 40°C, cooled to room temperature and hybridization was carried out at 40°C for 2 h in an InSlide Out hybridization oven (Boekel, Pennsylvania). The negative control consisted of the probe diluent only. One dot per every 10 cells displaying background staining at 20× magnification was considered acceptable according to the manufacturer. Slides were counterstained with Sudan Black B for 30 s, rinsed clear with deionised water and mounted with ProLong Gold Antifade Mountant (Thermo Fisher Scientific, Carlsbad, California).

### 2.3 Statistical Modelling

Individual fish records were used to evaluate HPML prevalence among adult smallmouth bass. All TL data were aggregated to 25‐mm TL bins due to data reporting discrepancies among fish collections. Each fish was assigned a TL measurement at the minimum value for each TL bin (i.e., a 489‐mm fish was categorised as 475 mm) and assigned a response value (*y*) of 1 (HPML presence) or 0 otherwise. Locational (site), temporal (Julian date) and environmental (water temperature) variables from the respective collections were linked to each fish record.

A Bayesian hierarchical logistical regression model was fitted to investigate how the predictor variables affected the prevalence of HPMLs among adult smallmouth bass. The model included the random effects of site and year and fixed effects of water temperature, TL bin, Julian date, and a water temperature by TL bin interaction. The interaction was included because exploratory analyses suggested seasonal differences in length‐frequency distribution and prevalence. The addition of random effects accounted for the nested nature of the data (i.e., multiple years and/or sites of collection) and lack of independence from samples collected at the same site and/or in the same year. The hierarchical logistic regression model was as follows:

Overall model statement:

(1) $$\begin{array}{c} \mathrm{Pr}\;\left(y_{i}=1\right)={\text{logit}}^{-1}(\;\beta_{0}+\beta_{1}*{\text{Temp}}_{i}+\beta_{2}*{\text{TLbin}}_{i}+\beta_{3}*{\text{Juliandate}}_{i}\\ \;+\beta_{4}*{\text{Temp}}_{i}:{\text{TLbin}}_{i}+\delta_{j\left[i\right]}+\varphi_{k\left[i\right]}),\text{for}\;i\;\text{in}\;1,\ldots \ldots .,16,130\;\text{observations} \end{array}$$

Site‐level:

(2) $$\delta_{j}\sim N\left(0,\sigma_{\delta }^{2}\right),\text{for}\;j\;\text{in}\;1\ldots ..,20\;\text{sites}$$

Year‐level:

(3) $$\varphi_{k}\sim N\left(0,\sigma_{\varphi }^{2}\right),\text{for}\;k\;\text{in}\;1\ldots ,11\;\text{years}$$

where $y_{i}$ = 1 represents a fish with HPML present and 0 indicates absence of HPMLs, $\;\beta_{0}$ is the model intercept, $\;\beta_{1-4}$ are regression coefficients for fixed effects in the model which corresponded to individual fish records and include water temperature at the time of collection $\;{\text{Temp}}_{i}$, TL bin $\;{\text{TLbin}}_{i}$, Julian date ${\text{Juliandate}}_{i}$, and an interaction between water temperature and TL bin ${\text{Temp}}_{i}*{\text{TLbin}}_{i}$. Random site $\delta_{j}$ (Equation 2) and year $\varphi_{k}$ (Equation 3) effects were assumed to be normally distributed with a mean of 0 and a variance of $\sigma_{\delta }^{2}$ and $\sigma_{\varphi }^{2}$, respectively. The model was fitted using the rstanarm package (Goodrich et al. 2024) in the R programming environment called from RStudio (Posit Team 2023). Water temperature, TL bin and Julian date were standardised prior to analysis. The model included one MCMC chain and ran for 2000 MCMC iterations with 1000 iterations discarded as burn‐in. Model convergence was evaluated using the Gelman Rubin $\hat{R}$. Posterior distributions were summarised for fixed and random effects with the posterior mean and 95% credible interval reported unless otherwise specified. For the temperature and length interaction, model results are reported for the average size fish in the dataset (224 mm) and fish ±1 SD in size (125 and 322 mm). Furthermore, these length categorizations align with age‐0 fish (125 mm), the transition between juvenile and young adult fish (224 mm), and adult fish (322 mm) based on previous studies in the Susquehanna River Basin (Li et al. 2018). If the 95% credible interval for a fixed effect overlapped 0, it was deemed not statistically significant. When this occurred, the posterior probability that the effect was in the direction of the posterior mean was also calculated to provide additional information about the potential ecological significance of the effect.

## 3 Results

**FIGURE 2:**
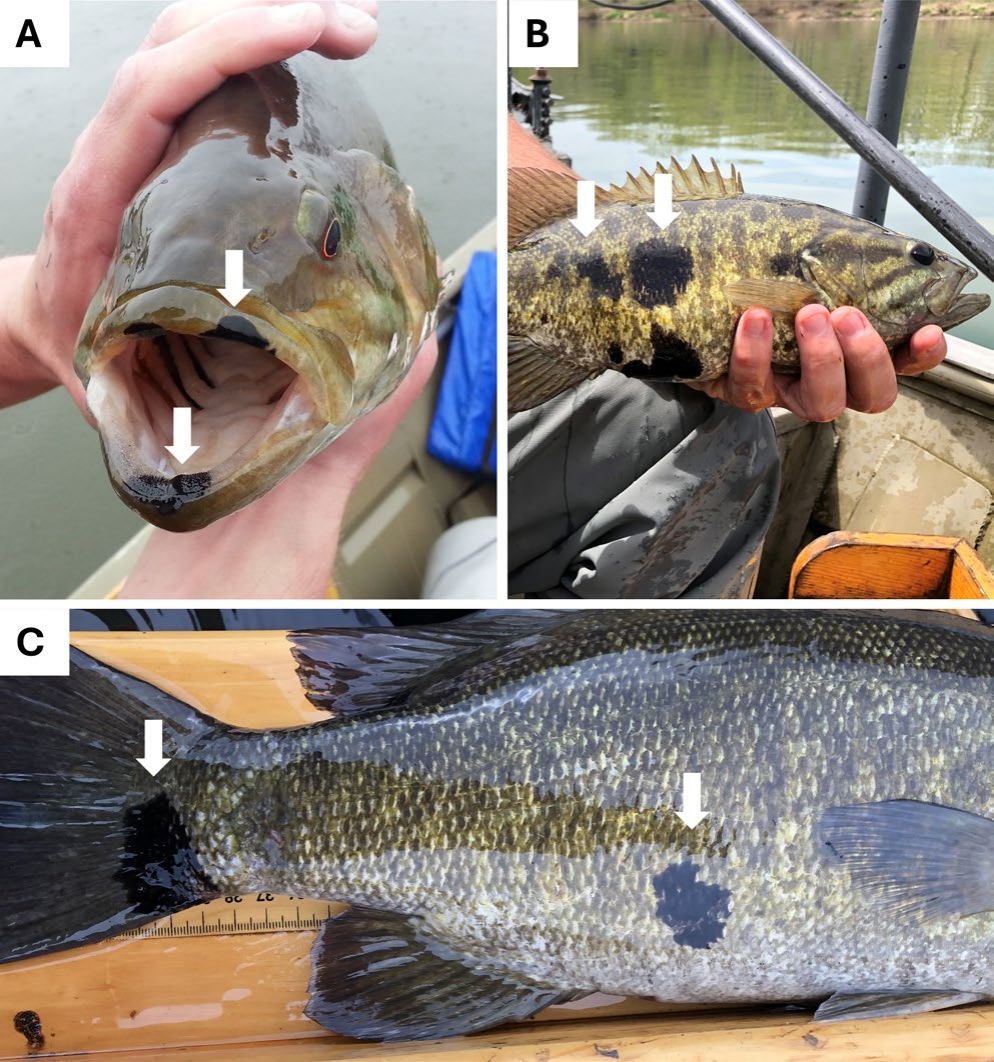
Visual representation of hyperpigmented melanistic lesions (HPMLs) on smallmouth bass from the Susquehanna River Basin, Pennsylvania. Arrows point to examples of HPMLs on smallmouth bass collected. Lesions were typically encountered on the mouth (A), body surface (B), and fins (C; also shown on the body).

**FIGURE 3:**
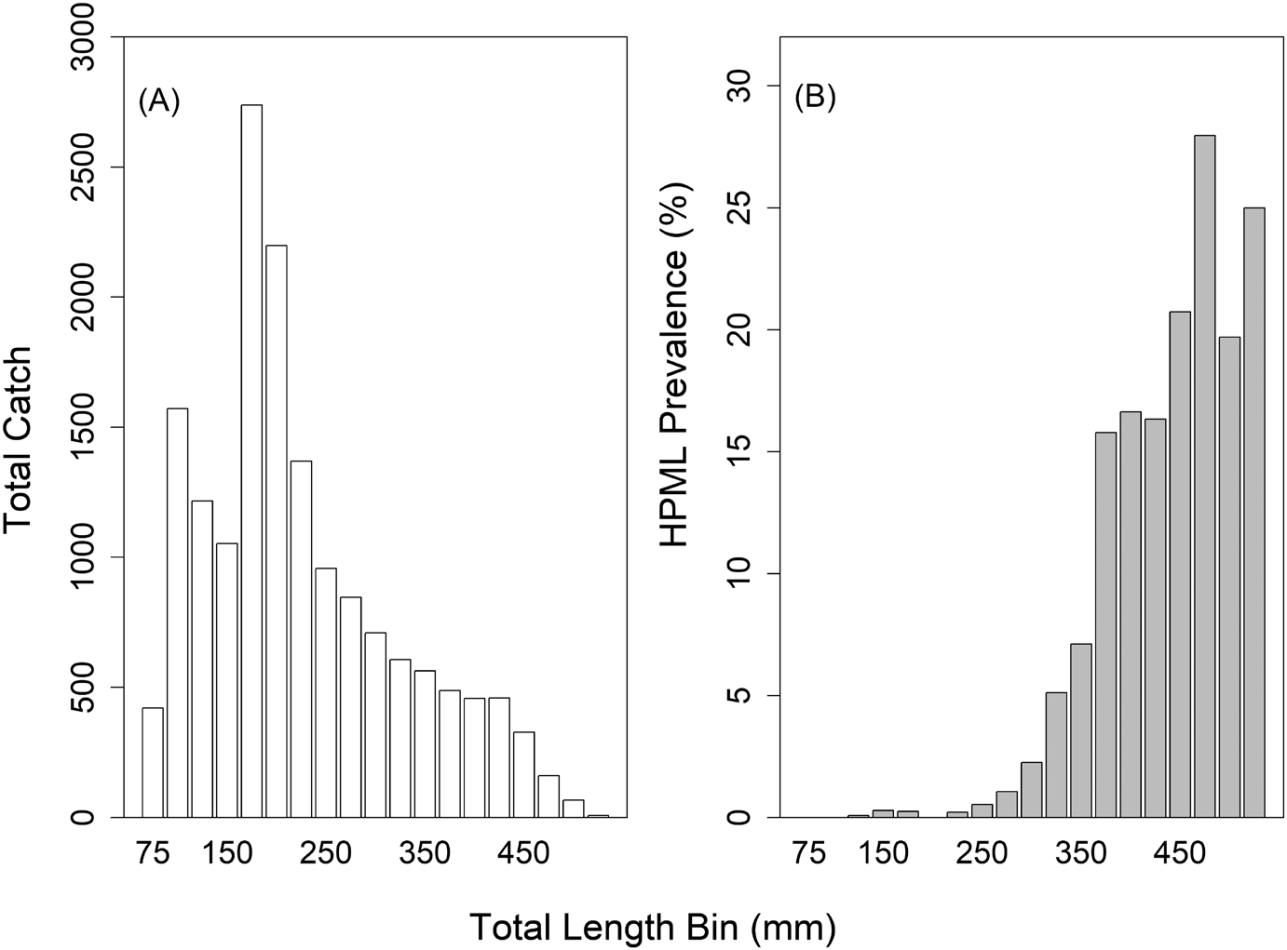
The total catch of smallmouth bass in the Susquehanna River Basin, Pennsylvania between 2012 and 2022 grouped into 25 mm total length (TL) bins (A) and the hyperpigmented melanistic lesion (HPML) prevalence across those same TL bins (B). Total length ranged from the 75 mm bin to the 525 mm bin.

A total of 16,220 smallmouth bass were collected across 20 sites in the Susquehanna River Basin from 130 surveys between 2012 and 2022. Across sampling years, sample size ranged from 361 fish in 2018 to 2921 fish in 2016. The average number of fish caught in a year was 1475 ± 728 individuals. Seventeen sites had repeat visits including four sites having 10 or more collections (Table 1). Fish lengths ranged from the 75‐mm TL bin to the 525‐mm TL bin. The average length collected was 224 ± 99 mm (mean ± SD). Overall, the prevalence of HPMLs during the study period was 2.9% (471/16,220). Visually, HPMLs were irregularly shaped, dark pigmented areas, noted on the external body surface, fins, and mouth (Figure 2). The lesions varied in size and shape ranging from a single focal lesion to multiple lesions covering large areas of the body. Variability in prevalence of HPMLs was documented with respect to fish size, site, year, and temperature at collection. Lesions were more commonly encountered on longer fish and rarely found on fish less than stock size (180 mm; Gabelhouse 1984) with only four fish < 175 mm having HPMLs (Figure 3). Smallmouth bass in the 425‐mm or longer TL bins had HPML prevalence of ≥ 19%, whereas fish in the 275‐mm or less TL bin had prevalence < 2%.

**TABLE 1:** Prevalence of hyperpigmented melanistic lesions (HPMLs) from smallmouth bass collections completed at twenty sampling sites in the Susquehanna River Basin, Pennsylvania between 2012 and 2022.

Site	Latitude	Longitude	Sampling years	Visits	Number HPML	Total catch	% HPML (mean ± SD)
Juniata River							
Mapleton	40.393921	−77.938779	2012–2017, 2019–2020	9	16	973	1.5 ± 1.4
Shawmut	40.370424	−77.811019	2017	1	8	255	3.1
Newton Hamilton	40.391579	−77.834305	2012–2017, 2019–2020	10	31	2030	1.3 ± 1.9
Granville	40.556519	−77.596697	2016–2017, 2019	3	5	392	1.4 ± 0.8
Lewistown Narrows	40.604576	−77.468873	2012–2014, 2016, 2020	5	3	2519	0.2 ± 0.5
Muskrat Springs	40.534197	−77.299725	2012–2013, 2017, 2019–2020	5	3	235	1.5 ± 1.4
Thompsontown	40.553633	−77.236576	2012–2017, 2019–2020	8	4	1199	0.2 ± 0.5
Greenwood	40.535772	−77.147738	2012–2017, 2019–2020	8	6	710	0.7 ± 1.3
Amity Hall	40.446486	−77.020447	2012–2013, 2015, 2016, 2020, 2022	6	30	678	5.1 ± 7.9
Susquehanna River							
Tunkhannock	41.535125	−75.95178	2021–2022	4	50	426	19.1 ± 13.7
Shady Nook	40.834426	−76.824032	2014–2015, 2017, 2019, 2022	8	85	757	11.4 ± 6.5
Port Trevorton	40.709594	−76.858351	2012	1	7	49	14.3
Liverpool	40.579254	−76.979	2012	1	3	35	8.6
Clemson Island	40.468987	−76.947789	2013–2020, 2022	10	14	1204	1.3 ± 1.4
Rockville	40.329736	−76.913013	2012–2020, 2022	17	95	2360	4.2 ± 5.0
West airview	40.272129	−76.910109	2019, 2022	3	44	169	24.6 ± 3.9
Dock Street	40.2442	−76.876668	2013–2020, 2022	9	8	773	1.4 ± 2.9
Turnpike	40.204618	−76.800208	2012–2015, 2017–2020, 2022	11	24	649	4.9 ± 6.1
Goldsboro	40.165869	−76.738201	2013–2017, 2019–2020	7	35	657	4.7 ± 2.9
Conowingo	39.811405	−76.308238	2020–2021	4	0	150	0 ± 0

*Note:* Sites are organised from upstream to downstream within each respective river (Juniata and Susquehanna rivers). Sampling years with data collected vary across sites, with most sites having repeat visits (Visits). The number of fish with HPMLs are summarised from all sampling events for each site. Total catch is summed over all sampling visits for each site. HPML prevalence represents the average HPML prevalence in percent over all sampling visits [mean ± standard deviation (SD)].

The Susquehanna River mainstem generally had a higher prevalence (5.0%, 365/7229) than the Juniata River (1.2%, 106/8991), however, site specific variability was observed (Table 1). Average HPML prevalence at a site ranged from 0 (Susquehanna Conowingo) to over 20% (Susquehanna West Fairview; Table 1). Repeated visits to sites also demonstrated variability in HPML prevalence among sampling events. The Susquehanna Rockville site which had 17 sampling events had HPML prevalence ranging from 0 (*n* = 3) to 16.7% with the average HPML prevalence of 4.2% ± 5.0% (Table 1).

**FIGURE 4:**
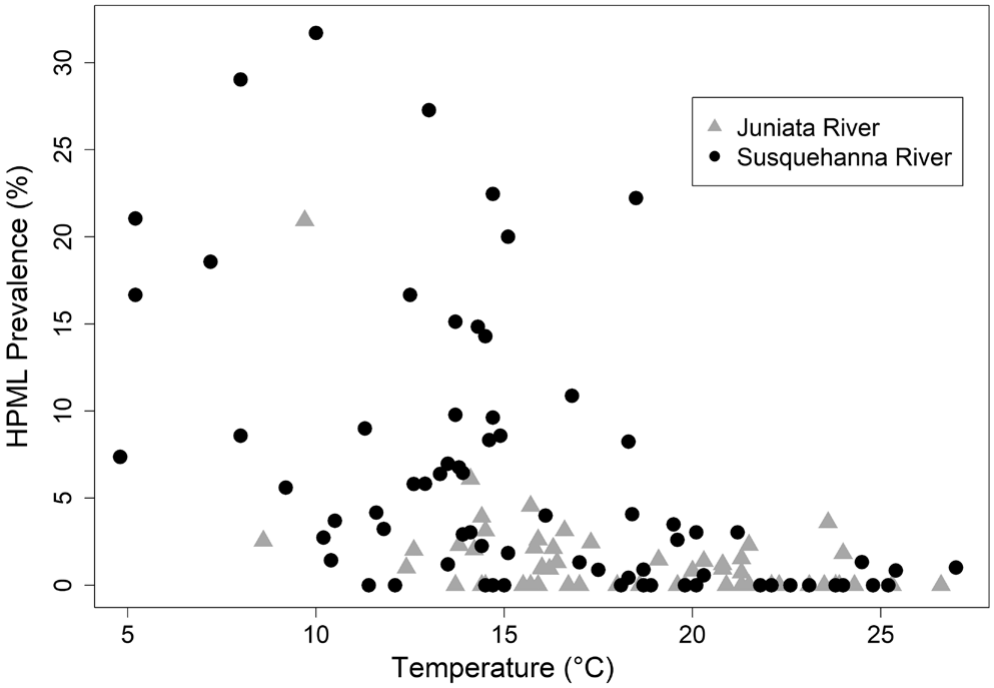
The water temperature (°C) at the time of smallmouth bass collection in the Susquehanna River (black circle) and Juniata River (grey triangle) and the hyperpigmented melanistic lesion (HPML) prevalence (%) for each sampling event.

Fish collections occurred during multiple sampling months (March, April, May, June, September, October and November) and across a range of temperatures (4.8°C–27.0°C). Most fish surveys occurred during the months of September (*n* = 41) and October (*n* = 67). The average water temperature across all collections was 17.0°C ± 4.9°C (mean ± SD). Prevalence of HPMLs exceeded 15% in 12 surveys with 10 of those occurring at temperatures < 15°C (Figure 4). The three coldest months of collection on average were March (mean ± SD [range]: 7.3°C [4.8–9.7], *n* = 2]), April [(9.6°C ± 2.6°C [7.2–13.0], *n* = 4) and November (9.0°C ± 2.8°C [5.2–12.6], *n* = 8). The warmest month of fish collection was June (23.5 ± 2.7 [18.9°C–25.4°C], *n* = 5). The month with the highest prevalence of HPMLs was April (26.4%, *n* = 80/303) followed by May (22.4%, *n* = 17/76), March (13.8%, *n* = 25/181) and then November (8.1%, *n* = 66/818).

### 3.1 Modelling Results

**FIGURE 5:**
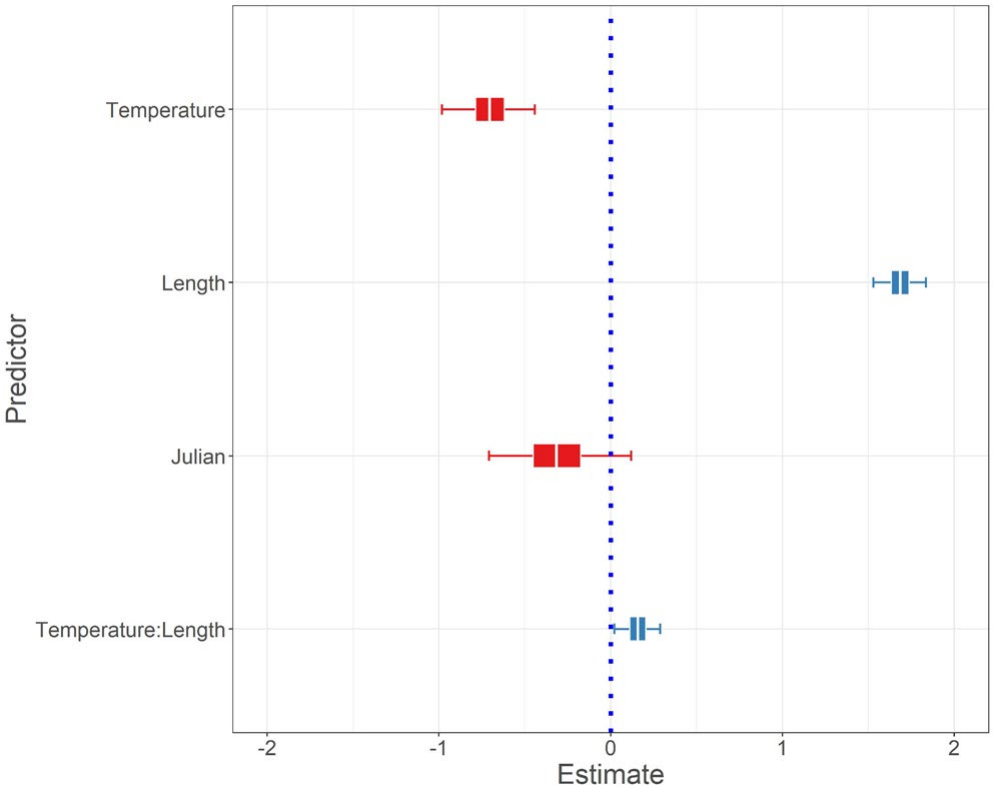
Estimates for predictors incorporated in the hierarchical logistic regression model including temperature, total length (TL) bin, Julian date, and a temperature by TL bin interaction. Posterior means, 50% credible intervals (inner bound), and 95% credible intervals (outer bound) are represented on the plot. Red coloration of box and whiskers denotes a negative effect and blue denotes a positive effect.

**FIGURE 6:**
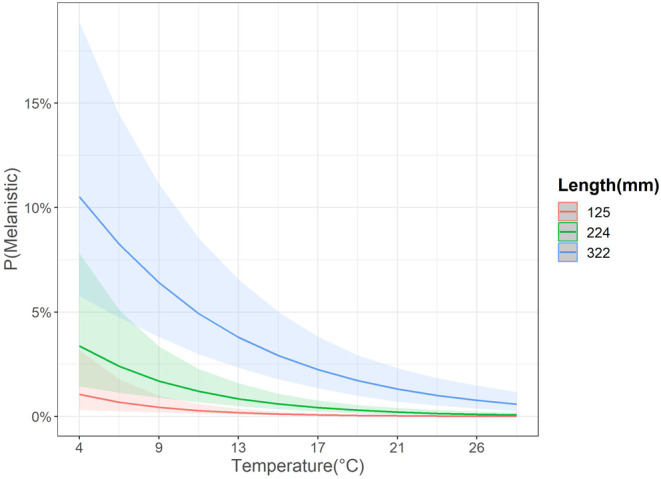
Model predicted estimates for the temperature by length interaction for prevalence of hyperpigmented lesions (HPMLs) in smallmouth bass. The green line and shading represent the relationship with fish of average length (224 mm). The red and blue lines and shading represent prevalence estimates for ± 1 SD of average length, 125 and 322 mm, respectively. Lines represent the posterior mean and shaded regions are the 95% credible interval.

**FIGURE 7:**
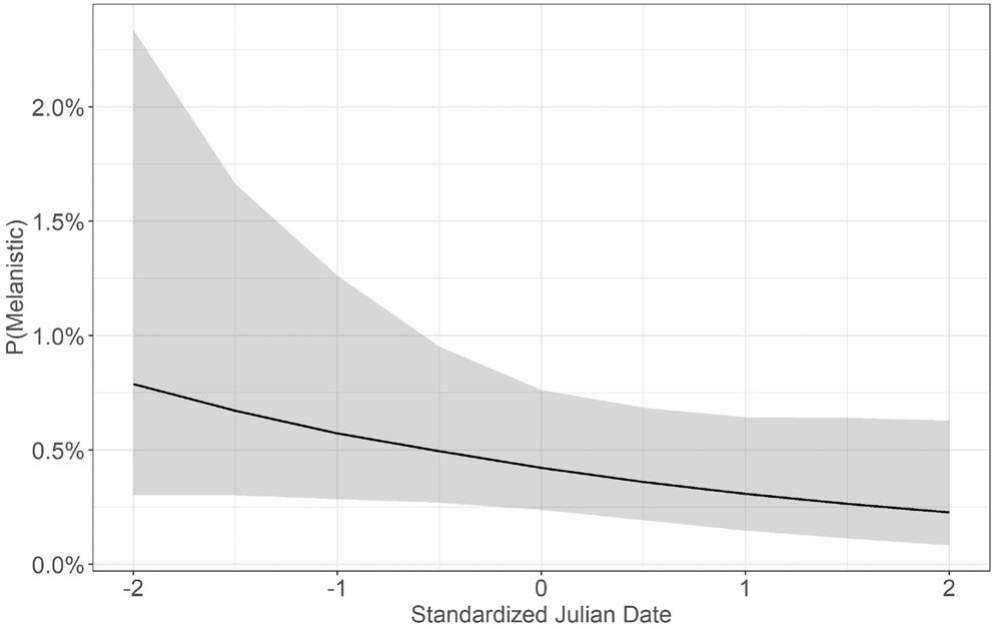
The estimated relationship between Julian date and prevalence of hyperpigmented melanistic lesions (HPMLs). The solid line represents the posterior mean, and the shaded area is the 95% credible interval.

**FIGURE 8:**
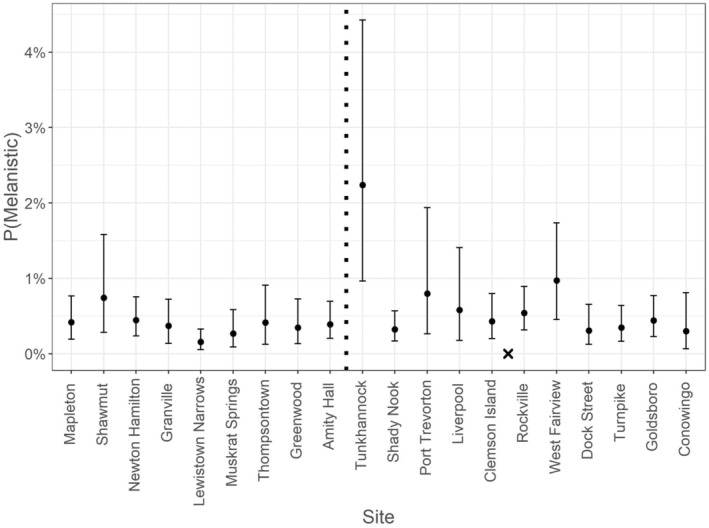
Modelling estimates for prevalence of hyperpigmented melanistic lesions (HPMLs) across the 20 sampling sites surveyed in the Susquehanna River Basin. Sites are separated (dashed vertical line) by Juniata River tributary sites on the left and Susquehanna River mainstem sites on the right of the vertical line. Sites are ordered from upstream to downstream in each respective section. The “X” between the Clemson Island and Rockville sites represents where the Juniata River confluences with the mainstem Susquehanna River. Solid circles are the posterior means and lines are the 95% credible intervals.

**FIGURE 9:**
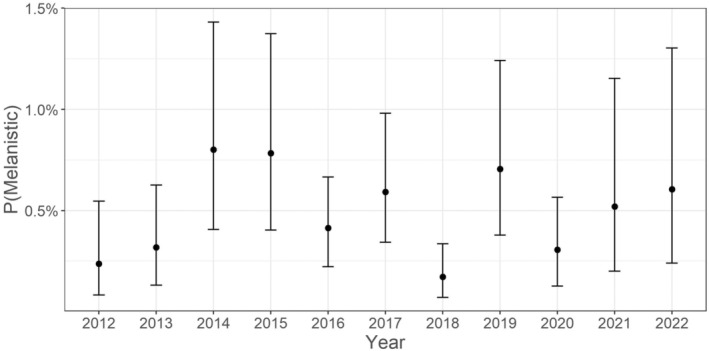
Modelling estimates for prevalence of hyperpigmented melanistic lesions (HPMLs) in smallmouth bass across sampling years (2012–2022) in the Susquehanna River Basin. Solid circles are the posterior means and lines are the 95% credible intervals.

Data from one sampling event were omitted (*n* = 90; Susquehanna Clemson Island, 2013) from the modelling effort due to missing temperature data, resulting in 16,130 fish included in the hierarchical logistic regression model. Mean estimated probability of HPML occurrence at average fish size, temperature, and Julian date was low (0.4% [0.2, 0.8]). Although the probability of HPML occurrence was greater at colder temperatures and for longer fish, there was a significant temperature and length (TL bin) interaction indicating that the probability of HPML occurrence was not similar across all sizes of fish at all temperatures [$\beta_{4}$; 0.16 (0.02, 0.29), Figure 5]. In fact, longer fish (322 mm) had a higher probability of HPML occurrence across all temperatures than did average length (224 mm) or shorter fish (125 mm) (Figure 6). For instance, the predicted probability of HPML occurrence at 4°C for a 322 mm fish was 10.5% (5.8, 18.9), whereas for a 224 mm and a 125 mm fish it was 3.4% (1.4, 7.8) and 1.1% (0.3, 3.2), respectively. Although lower predicted probability of HPML occurrence was documented at warmer temperatures (e.g., 26°C), 322 mm fish still had higher probability of occurrence (0.8% [0.4, 1.5]) than did 224‐mm (0.1% [0.0, 0.2]) or 125‐mm sized fish (0.01% [0.00, 0.04]). While not significant, Julian date had a 94.7% posterior probability of having a negative relationship with HPML prevalence [$\beta_{3}$; −0.32 (−0.71, 0.12); Figures 5 and 7], suggesting that HPMLs were less prevalent in surveys as the study duration progressed. Estimates for site and year HPML occurrence demonstrated annual and site‐specific variability (Figures 8 and 9). In the Susquehanna River, Tunkhannock, West Fairview, and Port Trevorton had the highest posterior mean probability of HPML occurrence with only Tunkhannock having an estimate above 1% (2.2% [1.0, 4.4]; Figure 8). In the Juniata River, the Shawmut site was estimated to have the highest probability of HPML occurrence (0.7% [0.3, 1.6]; Figure 8). The survey years with the highest predicted probability of HPML occurrence were 2014 and 2015, both with a posterior mean of 0.8% (Figure 9). The survey years with the lowest predicted probability of occurrence were 2012 and 2018, both with a posterior mean of 0.2% (Figure 9). Both site and year random effects had similar variability with respect to HPML prevalence [site $\sigma_{\delta }$; 0.51 (0.18, 1.16) and year σ_φ_; 0.50 (0.13, 1.41)].

### 3.2 Location of Virus Associated With HPMLs Using RNAscope

**FIGURE 10:**
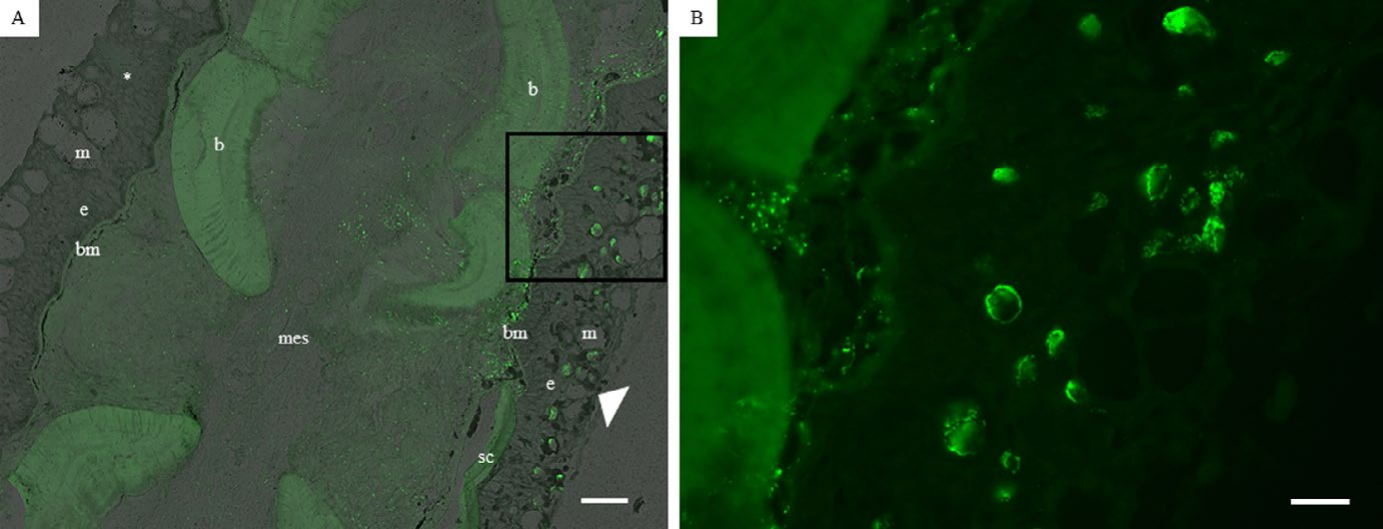
RNAscope results depicting the localization of the *Micropterus dolomieu* adomavirus 1 LO8 (MdA1 LO8) gene in a hyperpigmented melanistic lesion (HPML) in the fin collected from a Smallmouth Bass from the Susquehanna River at Shady Nook in April 2019. (A) Fluorescence with brightfield overlay image depicting the presence of MdA1 LO8 in the mesenchyme (mes), basement membrane (bm), and within melanocytes throughout the epidermis (e, arrowhead). In the opposing side, there is a lack of signal in normal epidermis (e). b = bone, m = mucous cells, sc = scale. Scale bar = 50 μm. (B) Higher magnification of the inset in (A) which shows individual viral nucleic acids. Scale bar = 20 μm.

In HPMLs, the RNAscope results showed that the MdA1 probe was associated with melanocytes in the epidermis and was not present in normal epidermal tissue (Figure 10A). In HPMLs, the virus was also present in the dermis and connective tissue. When the virus was present in sub‐epidermal layers, individual viral nucleic acids were scattered throughout. However, when it was present in the epidermis, it was directly associated with melanocytes and in most cases located within the nucleus and multiple viral nucleic acids were often present (Figure 10B). Many infected melanocytes were hypertrophied and typically had irregular, enlarged nuclei.

## 4 Discussion

We found that HPMLs occurred at a low prevalence rate on smallmouth bass from the Susquehanna River Basin. Prevalence of HPMLs, nonetheless, varied across sites, surveys, and years. Importantly, we found that the probability of HPML occurrence was affected by fish size and water temperature–with longer fish sampled during cooler water temperatures having a higher probability of HPML occurrence. Although it is not clear from this correlational study how these variables relate to the presence of the virus or the fish's response to infection, we posit a few potential mechanisms. Longer (i.e., older) bass may have different exposure histories due to behaviour and habitat usage. They may also have increased contaminant loads or exposure to other stressors. Water temperature can influence smallmouth bass ecology and the immune response to infectious agents (Abram, Dixon, and Katzenback 2017; Scharsack and Franke 2022).

Fish ecology and behaviour, such as life history traits or movement patterns, are difficult to quantify in relation to disease, and at the same time are important considerations for disease transmission and infection (Chapman et al. 2021). Smallmouth bass in riverine habitats exhibit complex seasonal movement patterns that vary among river systems and range from sedentary behaviours to long‐distance movements (Todd and Rabeni 1989; Langhurst and Schoenike 1990; Schall et al. 2019; McClure et al. 2020). In a radio‐telemetry study in the Susquehanna River Basin, long distance movements (> 10 km) were common, with fish congregating seasonally during late fall for overwintering when gonadal recrudescence would be occurring and during the spring for spawning (Schall et al. 2019). Movement and congregation during cold seasonal periods (i.e., temperatures less than 15°C) such as early spring, late fall, and winter also coincides with a higher incidence of HPMLs and could represent opportunities for disease transmission. Similarly, when stream temperatures were below 15.5°C during the fall in the Snake River, located in the western United States, smallmouth bass were no longer detected in shallow habitat and suspected to be in overwintering groups (Munther 1970). In the Susquehanna River Basin, more than 20 radio‐tagged fish were located within an impounded reach of the river for overwintering while stream temperatures were below 5°C (Schall et al. 2019); the same temperature range we would expect to encounter the highest HPML prevalence (Figure 4). Water temperature influences many behaviours in smallmouth bass such as reproductive ecology (Graham and Orth 1986), daily and seasonal movement patterns (Todd and Rabeni 1989) and in this study it was also a significant predictor of HPML occurrence.

Although we currently do not have information on viral replication and transmission pathways for the adomavirus associated with HPMLs, temperature is regarded as a regulator of viral diseases in fish, altering viral infection dynamics, immune response, and fish outcome (Walker and Winton 2010). Cold temperatures have been shown to modify and reduce both acquired and innate immune responses to viral infections, however, not all fish species or immune functions respond similarly (Scharsack and Franke 2022). In fact, some innate immune characteristics may not be modulated during cold temperatures, leading to reliance on responses such as inflammatory mechanisms (e.g., maintenance of IL‐1β, iNOS and TNF‐α gene transcripts at cold temperatures; Dios et al. 2010). Comparatively, in olive flounder *Paralichthys olivaceus* infected with *Hirame novirhabdovirus* (HIRRV), genes involved in viral recognition (i.e., RIG‐I like receptors) and protein stability/viral replication (i.e., heat shock proteins) were only upregulated at warmer temperatures (20°C) when compared to a colder temperature (10°C; Wang et al. 2020). Specific viruses are common or more severe in colder temperature ranges (Scharsack and Franke 2022). For example, mortality occurred in over 85% of flounder infected with HIRRV at 10°C, whereas most fish (> 85%) survived at 20°C in a laboratory study (Wang et al. 2020). Similarly, outbreaks of infectious haematopoietic necrosis virus (IHNV) in salmonid fishes are often at temperatures below 15°C, although there have been species‐ and environment‐dependent temperature deviations in infections described (Dixon et al. 2016). Although we do not have comparable information on temperature‐dependent infection of closely related viruses in *Adomaviradae*, water temperature in this study has emerged as an important consideration for HPML prevalence. Yet, temperature effects on viral infections may not be consistent across all seasons or with respect to stress‐inducing life history events (e.g., spawning). For example, higher incidences of viral hemorrhagic septicemia (VHS‐IVB) in smallmouth bass were documented during spawning conditions (i.e., 10°C–14°C) when compared to water temperatures that were similar, warmer, or colder throughout the year (Eckerlin et al. 2011). Similarly, colder water temperatures associated with a reduced fish immune response may present an opportunity for viral infection and the manifestation of lesions from the adomavirus associated with HPMLs in this study.

The response of melanin deposition in HPMLs may also be related to immune function influenced by colder temperatures. Melanin in this case may play a role as a protective mechanism when environmental stressors (lower temperature, increased contaminant loads) reduce the more classic immune response. Human melanocytes are found in the epidermis (Cichorek et al. 2013) and melanin within the epidermal melanocytes has classically been considered to have the primary and critical role of photoprotection by absorbing ultraviolet radiation (UVB). However, in humans a link between melanogenesis and innate immunity and a role for melanocytes in viral infections has been noted (reviewed by Koike and Yamasaki 2020). It has been suggested that melanin serves as a free radical scavenger in fishes (Agius and Agbede 1984) and is important in the production of antimicrobial compounds (Wolke 1992), particularly at low temperatures when enzymatic activity is restricted. This may also explain the increased prevalence of HPMLs at lower temperatures.

The effect of temperature was, however, dependent on fish length as longer, more mature fish were predicted to have higher HPML prevalence across all temperatures. There are a number of possible explanations for the length‐specific differences in HPML prevalence, including behaviour/habitat usage and chemical contaminant exposures or bioaccumulations that increase with age and adversely affect the immune response. Mature adult and juvenile bass utilise different habitats, limiting overlap and viral transmission. Wolf, Mollenhauer, and Brewer (2019) suggest that age‐0 smallmouth bass overwintered in different habitats than age‐1+ individuals. Lifetime exposure histories through habitats occupied and age can also influence concentrations of certain contaminants. Bioaccumulative contaminants including mercury (Blazer et al. 2023) and per‐ and polyfluoroalkyl substances (PFAS; Blazer et al. 2021) have been documented in smallmouth bass from the Susquehanna River Basin. Hyperpigmentation in common dab *Limanda limanda* which ranges from patches to extensive, diffuse coverage has been documented for many years in the North Sea and adjacent areas (Lang et al. 2017). An increased prevalence with dab length has been observed (Grütjen et al. 2013), yet to date no infectious agents have been identified (Noguera et al. 2013). Modelling monitoring data from multiple years and sites indicated cadmium, lead, and a chlorinated biphenyl (CB118), as well as population genetic structure, were good predictors of hyperpigmentation (Tysklind et al. 2013). Thus, increased prevalence of HPMLs in older fish could be related to environmental exposures and varied effects on immune function leading to differences in disease (viral) susceptibility.

**FIGURE 11:**
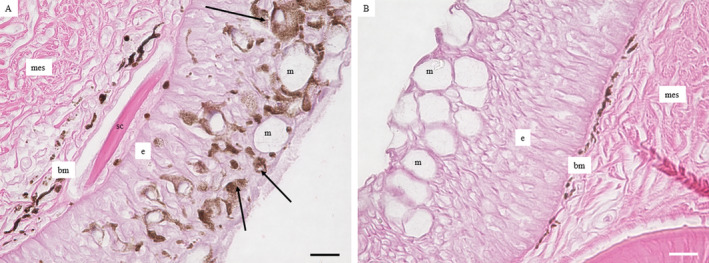
(A) A fin of a smallmouth bass with a hyperpigmented melanistic lesion (HPML) with melanocytes (arrows) that have migrated from the dermis below the basement membrane (bm) into the epidermis (e). (B) Normal skin from the opposing side of the fin from the same fish. m = mucous cells, mes = mesenchyme, sc = scale. Scale bar = 20 μm.

In smallmouth bass, the histological features of HPMLs have been well characterised by Blazer et al. (2020) and Young (2018). In brief, melanocytes, sometimes hyperplastic and pleomorphic, appear to both migrate to and proliferate in the epidermis (Figure 11A). Normal skin melanocytes reside beneath the basement membrane in the dermis (Figure 11B). Previous studies in fish species have observed the relationship between melanin and cellular damage. Early work demonstrated the occurrence of melanophores in experimental wounds of goldfish *Carassius auratus* (Smith 1931). More recently studies with the zebrafish *Danio rerio* model demonstrated melanocytes migrating to sites of wounds or implanted beads and wrapping around implanted beads. It was further demonstrated the migration of melanocytes is a secondary inflammatory cell‐mediated response (Lévesque et al. 2013). Melanocytes have also been shown to migrate from the dermis to the epidermis in response to numerous stressors including acidified water (Iger and Wendelaar Bonga 1994) and river water with a complex mixture of contaminants (Iger, Jenner, and Wendelaar Bonga 1994).

Hyperpigmented melanistic lesions on smallmouth bass from the Susquehanna River Basin were previously associated with a novel adomavirus (MdA1). Identification of viral helicase gene transcripts in HPMLs led to the discovery of an undescribed virus, which aligned the closest with an adomavirus found in white‐spotted wedgefish *Rhynchobatus djiddensis* (Blazer et al. 2020). Our study provides additional support for a viral infection through the detection of MdA1 adomavirus viral nucleic acids in HPMLs (MdA1 LO8; NCBI Accession No. MZ673484.1) with RNAscope. However, more in depth studies using RNAscope are required to document the progression of the cellular response during the infection. Future studies that use genomics and in situ hybridization could enhance understanding of the melanocyte response to this virus. In addition, a deeper understanding of the influence and timing of temperature changes or of exposure to low temperature on the viral progression and infection dynamics is needed. With RNAscope, individual MdA1 viral nucleic acids appeared to be scattered throughout the sub‐epidermal layers, however, within the epidermis were incorporated into the nuclei of the melanocytes and was not observed in normal epidermal tissue. It is currently unknown whether phagocytosis or uptake of the virions triggers migration of infected melanocytes into the epidermis or virions within the epidermis trigger melanocyte proliferation or both.

The observation of black pigmentation and melanocytes in association with infectious agents in fishes is not new. “Black spot disease” is a condition observed on numerous fish species which is caused by melanin deposited around encysted metacercariae of multiple genera of digenetic trematodes (Hsiao 1941; Hoffman and Putz 1965; Steedman 1991). It is interesting to note that dark pigmentation has been observed in salmon tissues containing piscine orthoreovirus (Bjørgen et al. 2019; Malik et al. 2021). However, in these cases the melanistic response was associated with chronic granulomatous inflammation containing melano‐macrophages which were not noted in smallmouth bass skin. Transcript changes showed arginase‐2 was linked to the melanized areas and macrophages in salmon (Malik et al. 2021). In smallmouth bass, arginase‐1 was not up‐regulated in melanistic areas, rather transcripts associated with melanocytes and melanin synthesis, such as tyrosinase (*tyr*), melanocyte premelanosome protein‐like (*pmel*) and micropthalmia‐associated transcription factor (*mitf*) were upregulated (Blazer et al. 2020). The expression of tyrosinase genes has also been shown to occur in melanomacrophages with chronic inflammation (Larsen et al. 2012). Further investigation into gene regulation can help identify the cellular mechanisms occurring alongside melanin deposition with infectious agents.

It is not clear how other infections or conditions (both relating to fish health and the environment) may contribute to HPML prevalence and if the accumulation of these effects could lead to different fish health outcomes. Similarly, our work suggested a decline in HPML prevalence over the duration of the study, yet it is important to note that the overall prevalence was low throughout the study duration. In general, a robust set of ecological and fish‐specific attributes are beneficial in understanding disease dynamics and prevalence. This larger, more comprehensive dataset identified some relationships, such as length of affected fish, that previous analyses identified (Blazer et al. 2020) while others, such as temperature, were not apparent until the size of the dataset increased. Disease screening of a visible lesion or anomaly could also provide an opportunity for quick recognition of future fish health conditions while fish are being assessed. To date, there is no evidence that HPMLs in smallmouth bass are associated with mortality. However, melanistic lesions are readily visible to the public and anglers which could cause concern about fish health or a negative perception of the fishery. Importantly, longer smallmouth bass, the portion of the population targeted by anglers, are most likely to be encountered with higher prevalence of HPMLs. Although, overall HPML prevalence is low across the population, it may not appear as such to the public. Having fish population data, fish health data and environmental variables together may help to elucidate patterns in fish populations quickly and effectively to facilitate communication on population health and status with the public.

## Author Contributions

**Megan K. Schall:** conceptualization, writing – original draft, methodology, writing – review and editing, formal analysis, visualization, data curation, validation. **Geoffrey D. Smith:** conceptualization, investigation, funding acquisition, writing – original draft, writing – review and editing, visualization, methodology, data curation. **Vicki S. Blazer:** writing – original draft, methodology, writing – review and editing, visualization, funding acquisition, investigation. **Heather L. Walsh:** methodology, investigation, writing – review and editing, formal analysis, writing – original draft. **Tyler Wagner:** formal analysis, methodology, writing – original draft, writing – review and editing, visualization, validation.

## Conflicts of Interest

The authors declare no conflicts of interest.

## Data Availability

Data used in this project are available through the state management agency (Pennsylvania Fish and Boat Commission) upon request. The code for statistical modeling is available through the corresponding author (M.Schall).
